# Supplementary material for: Comparative genomics provides new insights into the diversity, physiology, and sexuality of the only industrially exploited tremellomycete: Phaffia rhodozyma
Source: BMC Genomics. 2016 Nov 9;17:901. doi: 10.1186/s12864-016-3244-7 (PMC5103461; doi:10.1186/s12864-016-3244-7)
Supplement: Additional file 6: — List of orphan genes with links to PFAM (related to Additional file 1: Table S1). (ZIP 1428 kb) [file 12864_2016_3244_MOESM6_ESM.zip › PFAM_UNIQUE/G01428_P.html]

#                                                                            --- full sequence --- -------------- this domain -------------   hmm coord   ali coord   env coord
  
# target name        accession   tlen query name           accession   qlen   E-value  score  bias   #  of  c-Evalue  i-Evalue  score  bias  from    to  from    to  from    to  acc description of target
  
#------------------- ---------- ----- -------------------- ---------- ----- --------- ------ ----- --- --- --------- --------- ------ ----- ----- ----- ----- ----- ----- ----- ---- ---------------------
  
G01428\_P             -            457 Couple\_hipA          PF13657.1     99      0.01   14.8   0.0   1   1   4.7e-06     0.028   13.4   0.0     3    69   185   253   184   272 0.89 - HipA N-terminal domain  
G01428\_P             -            457 HBS1\_N               PF08938.5     79    0.0056   15.5   0.0   1   2       0.3     9e+02   -1.2   0.0    19    42   221   244   218   247 0.87 - HBS1 N-terminus  
G01428\_P             -            457 HBS1\_N               PF08938.5     79    0.0056   15.5   0.0   2   2   9.6e-06     0.029   13.2   0.0    14    42   392   420   369   427 0.89 - HBS1 N-terminus
